# Supplementary material for: Schizophrenia-associated genomic copy number variants and subcortical brain volumes in the UK Biobank
Source: Mol Psychiatry. 2019 Jan 24;25(4):854–62. doi: 10.1038/s41380-019-0355-y (PMC7156345; doi:10.1038/s41380-019-0355-y)
Supplement: Supplementary file 1 — Supplemental material [file 41380_2019_355_MOESM1_ESM.docx]

Supplementary material

Supplemental Table 1. List of schizophrenia associated CNVs with critical region coordinates, calling criteria and number of genes hit. Schizophrenia (SZ) relative risk estimates for each CNV were taken from Rees *et al* 2016. PSD = Post-synaptic density, WBS = Williams-Beuren syndrome, PWS/AS = Prader-Willi/Angelman syndromes.

| **Syndrome** | **Locus** | **Critical/Unique**  **Sequence Region (hg19)** | **N Bialleleic**  **Axiom probes** | **PSD Genes (from 685 PSD genes)** | **Calling criteria** | **N genes** | **SZ relative risk** |
| --- | --- | --- | --- | --- | --- | --- | --- |
| 1q21.1del | 1q21.1 | chr1:146,527,987-147,394,444 | 289 |  | Size >50% of critical region | 9 | 5.6 |
| 1q21.1dup | 1q21.1 | chr1:146,527,987-147,394,444 | 289 |  | Size >50% of critical region | 9 | 2.1 |
| *NRXN1*del | 2p16.3 | chr2:50145643-51259674 | 235 | *NRXN1* | Exonic deletions | 1 | 4.2 |
| 3q29del | 3q29 | chr3:195,720,167-197,354,826 | 627 | *DLG1* | Size >50% of critical region | 28 | 13.5 |
| WBSdup | 7q11.23 | chr7:72,744,915-74,142,892 | 349 | *STX1A* | Size >50% of critical region | 26 | 4.3 |
| 15q11.2del BP1-BP2 | 15q11.2 | chr15:22,805,313-23,094,530 | 145 | *CYFIP1* | Size >50% of critical region | 5 | 1.7 |
| PWS/ASdup | 15q11-q13 | chr15:22,805,313-28390339 | 1807 | *CYFIP1* | Full critical region, ~4Mbp | 116 | 29.2 |
| 15q13.3del BP4-BP5 | 15q13.3 | chr15:31,080,645-32,462,776 | 466 |  | Size >50% of critical region | 8 | 4.2 |
| 16p13.11dup | 16p13.11 | chr16:15,511,655-16,293,689 | 349 | *MYH11* | Size >50% of critical region | 7 | 1.7 |
| 16p12.1del (520kb) | 16p12.1 | chr16:21,950,135-22,431,889 | 168 | *UQCRC2* | Size >50% of critical region | 8 | 3.4 |
| 16p11.2dup (593kb) | 16p11.2 | chr16:29,650,840-30,200,773 | 193 | *ALDOA,CORO1A,MAPK3,TAOK2* | Size >50% of critical region | 30 | 8.6 |
| 22q11.2del | 22q11.2 | chr22:19,037,332-21,466,726 | 843 | *PI4KA,SEPT5* | Size >50% of critical region | 61 | 21.6 |

Supplemental Table 2. Summary statistics from linear regression analysis of the effect of SZ-CNV carrier status on the 15 subcortical brain volumes (subsample of non-carriers to carriers matched by age)

| Subcortical volume | non-CNV  carriers | SZ-CNV  carriers | B | SE | p-value | FDR |
| --- | --- | --- | --- | --- | --- | --- |
| Thalamus (left) | 7314 | 47 | -0.18 | 0.09 | 0.046 | 0.085 |
| Thalamus (right) | 7320 | 47 | -0.22 | 0.09 | 0.014 | ***0.043*** |
| Caudate (left) | 7292 | 48 | -0.09 | 0.12 | 0.448 | 0.560 |
| Caudate (right) | 7302 | 48 | -0.07 | 0.12 | 0.540 | 0.582 |
| Putamen (left) | 7320 | 48 | -0.33 | 0.11 | 0.002 | ***0.014*** |
| Putamen (right) | 7315 | 47 | -0.24 | 0.11 | 0.027 | 0.069 |
| Pallidum (left) | 7257 | 46 | -0.26 | 0.13 | 0.039 | 0.083 |
| Pallidum (right) | 7264 | 47 | -0.37 | 0.12 | 0.003 | ***0.014*** |
| Hippocampus (left) | 7273 | 46 | -0.15 | 0.13 | 0.245 | 0.334 |
| Hippocampus (right) | 7256 | 47 | -0.50 | 0.13 | <0.001 | ***0.001*** |
| Amygdala (left) | 7327 | 46 | -0.03 | 0.14 | 0.840 | 0.840 |
| Amygdala (right) | 7314 | 47 | -0.19 | 0.14 | 0.162 | 0.244 |
| Accumbens (left) | 7293 | 47 | -0.25 | 0.13 | 0.052 | 0.087 |
| Accumbens (right) | 7316 | 48 | -0.36 | 0.13 | 0.004 | ***0.016*** |
| Ventricular volume | 7213 | 45 | 0.08 | 0.13 | 0.543 | 0.582 |

Supplemental Table 3. Summary statistics from linear regression analysis of the effect of 15q11.2deletion carrier status on the 15 subcortical brain volumes

| Subcortical volume | non-CNV carriers | 15q11.2del  carriers | B | SE | p-value | FDR |
| --- | --- | --- | --- | --- | --- | --- |
| Thalamus (left) | 8511 | 23 | -0.17 | 0.13 | 0.180 | 0.338 |
| Thalamus (right) | 8515 | 23 | -0.26 | 0.13 | 0.042 | 0.105 |
| Caudate (left) | 8501 | 23 | -0.09 | 0.17 | 0.617 | 0.712 |
| Caudate (right) | 8514 | 23 | -0.03 | 0.17 | 0.844 | 0.844 |
| Putamen (left) | 8535 | 23 | -0.36 | 0.16 | 0.022 | 0.082 |
| Putamen (right) | 8525 | 23 | -0.19 | 0.15 | 0.219 | 0.365 |
| Pallidum (left) | 8472 | 23 | -0.26 | 0.18 | 0.138 | 0.295 |
| Pallidum (right) | 8480 | 23 | -0.36 | 0.17 | 0.036 | 0.105 |
| Hippocampus (left) | 8484 | 23 | -0.46 | 0.18 | 0.013 | 0.066 |
| Hippocampus (right) | 8460 | 23 | -0.62 | 0.18 | 0.001 | ***0.009*** |
| Amygdala (left) | 8540 | 22 | -0.12 | 0.20 | 0.561 | 0.701 |
| Amygdala (right) | 8526 | 23 | -0.20 | 0.20 | 0.325 | 0.451 |
| Accumbens (left) | 8505 | 22 | -0.18 | 0.19 | 0.330 | 0.451 |
| Accumbens (right) | 8519 | 23 | -0.50 | 0.18 | 0.006 | ***0.047*** |
| Ventricular volume | 8440 | 21 | 0.06 | 0.18 | 0.747 | 0.800 |

Supplemental Table 4. Summary statistics from linear regression analysis of the effect of carrying any SZ-CNV other than 15q11.2deletion status on the 15 subcortical brain volumes

| Subcortical volume | Non-CNV carriers | non15q11.2del  CNV-carriers | B | SE | p-value | FDR |
| --- | --- | --- | --- | --- | --- | --- |
| Thalamus (left) | 8511 | 24 | -0.18 | 0.13 | 0.158 | 0.314 |
| Thalamus (right) | 8515 | 24 | -0.17 | 0.12 | 0.167 | 0.314 |
| Caudate (left) | 8501 | 25 | -0.11 | 0.16 | 0.522 | 0.603 |
| Caudate (right) | 8514 | 25 | -0.12 | 0.17 | 0.470 | 0.587 |
| Putamen (left) | 8535 | 25 | -0.31 | 0.15 | 0.040 | 0.228 |
| Putamen (right) | 8525 | 24 | -0.28 | 0.15 | 0.061 | 0.228 |
| Pallidum (left) | 8472 | 23 | -0.26 | 0.18 | 0.136 | 0.314 |
| Pallidum (right) | 8480 | 24 | -0.36 | 0.17 | 0.033 | 0.228 |
| Hippocampus (left) | 8484 | 23 | 0.18 | 0.18 | 0.341 | 0.465 |
| Hippocampus (right) | 8460 | 24 | -0.35 | 0.18 | 0.048 | 0.228 |
| Amygdala (left) | 8540 | 24 | 0.05 | 0.19 | 0.799 | 0.799 |
| Amygdala (right) | 8526 | 24 | -0.20 | 0.19 | 0.303 | 0.455 |
| Accumbens (left) | 8505 | 25 | -0.28 | 0.17 | 0.105 | 0.314 |
| Accumbens (right) | 8519 | 25 | -0.19 | 0.17 | 0.266 | 0.443 |
| Ventricular volume | 8440 | 24 | 0.09 | 0.17 | 0.593 | 0.635 |

Supplemental Table 5. Summary statistics from linear regression analysis of the effect of 16p13.11duplication carrier status on the 15 subcortical brain volumes

| Subcortical volume | Non-CNV carriers | 16p13.11dup  CNV-carriers | B | SE | p-value | FDR |
| --- | --- | --- | --- | --- | --- | --- |
| Thalamus (left) | 8511 | 9 | 0.03 | 0.21 | 0.885 | 0.885 |
| Thalamus (right) | 8515 | 9 | 0.05 | 0.20 | 0.789 | 0.885 |
| Caudate (left) | 8501 | 9 | 0.33 | 0.27 | 0.233 | 0.672 |
| Caudate (right) | 8514 | 9 | 0.28 | 0.28 | 0.301 | 0.672 |
| Putamen (left) | 8535 | 9 | -0.14 | 0.25 | 0.562 | 0.844 |
| Putamen (right) | 8525 | 8 | -0.04 | 0.26 | 0.872 | 0.885 |
| Pallidum (left) | 8472 | 8 | -0.32 | 0.30 | 0.283 | 0.672 |
| Pallidum (right) | 8480 | 8 | -0.36 | 0.29 | 0.218 | 0.672 |
| Hippocampus (left) | 8484 | 9 | 0.53 | 0.29 | 0.073 | 0.672 |
| Hippocampus (right) | 8460 | 9 | -0.37 | 0.29 | 0.194 | 0.672 |
| Amygdala (left) | 8540 | 9 | 0.31 | 0.31 | 0.324 | 0.672 |
| Amygdala (right) | 8526 | 8 | -0.31 | 0.34 | 0.358 | 0.672 |
| Accumbens (left) | 8505 | 9 | -0.12 | 0.29 | 0.683 | 0.885 |
| Accumbens (right) | 8519 | 9 | -0.20 | 0.29 | 0.494 | 0.824 |
| Ventricular volume | 8440 | 8 | -0.06 | 0.30 | 0.838 | 0.885 |

Supplemental Table 6. Summary statistics from linear regression analysis of the effect of 1q21.1duplication carrier status on the 15 subcortical brain volumes

| Subcortical volume | Non-CNV carriers | 1q21.1dup  CNV-carriers | B | SE | p-value | FDR |
| --- | --- | --- | --- | --- | --- | --- |
| Thalamus (left) | 8511 | 6 | -0.58 | 0.25 | 0.022 | 0.058 |
| Thalamus (right) | 8515 | 6 | -0.56 | 0.25 | 0.023 | 0.058 |
| Caudate (left) | 8501 | 6 | -0.85 | 0.33 | 0.011 | 0.054 |
| Caudate (right) | 8514 | 6 | -0.69 | 0.34 | 0.042 | 0.079 |
| Putamen (left) | 8535 | 6 | -0.89 | 0.30 | 0.003 | ***0.030*** |
| Putamen (right) | 8525 | 6 | -0.86 | 0.30 | 0.004 | ***0.030*** |
| Pallidum (left) | 8472 | 6 | -0.65 | 0.35 | 0.059 | 0.095 |
| Pallidum (right) | 8480 | 6 | -0.82 | 0.34 | 0.015 | 0.055 |
| Hippocampus (left) | 8484 | 5 | -0.37 | 0.40 | 0.344 | 0.430 |
| Hippocampus (right) | 8460 | 6 | -0.77 | 0.35 | 0.030 | 0.063 |
| Amygdala (left) | 8540 | 6 | -0.06 | 0.38 | 0.869 | 0.873 |
| Amygdala (right) | 8526 | 6 | 0.14 | 0.39 | 0.716 | 0.826 |
| Accumbens (left) | 8505 | 6 | -0.34 | 0.36 | 0.336 | 0.430 |
| Accumbens (right) | 8519 | 6 | 0.06 | 0.35 | 0.873 | 0.873 |
| Ventricular volume | 8440 | 6 | 0.63 | 0.34 | 0.063 | 0.095 |
